# Weekly simulation for an on call helicopter emergency medical crew: feasible or impossible?

**DOI:** 10.1186/1757-7241-23-S2-A23

**Published:** 2015-09-11

**Authors:** Per P Bredmose, Stephen Sollid

**Affiliations:** 1 Norwegian Air Ambulance Foundation, Drøbak, Norway; 2 Air Ambulance department, Prehospital Centre, Oslo University Hospital, Norway

## Background

In-situ training can be a time-effective way to give pre-hospital personnel an opportunity to train procedures and interventions as a team. We describe the feasibility of weekly simulation training for on-duty crews at Oslo, Norway, helicopter emergency medical service (HEMS).

## Methods

HEMS crews (doctor, HEMS crew member and pilot) were given the opportunity to do in-situ simulation during on call hours once a week. A simple mannequin and training equipment similar to equipment used in daily practice in the service were used. All training took place locally, either indoors or outdoors near the base. A single facilitator conducted all training during daytime. Scenarios were changed to allow all doctors to go through a set of themes during one year, and to give variability for the rest of the crew. We recorded data on the number of simulations that were carried out and time consumption, and collected data from the participating crews on a feedback form.

## Results

During one year 52 % of the planned simulations were completed. The major reasons for not performing training were missions. The median total time (and interquartile range (IQR)) for a complete simulation training epiosode was 65 min (58,74). The median score from the participants regarding “attitude to this kind of training” was 1 on a 7 pt. Likert scale (1= most positive score possible).

## Discussion

Weekly simulation provided a unique opportunity to train the whole crew in medical matters, team matters and decision-making. This form of training is cost effective because it takes place during working hours for the on-call crew. By training on-site with familiar operational equipment the HEMS rescue man and the pilot also get hands-on training and familiarisation with procedures and equipment.

## Conclusion

In situ simulation training during on-call hours is feasible in a busy HEMS service with no additional costs than for a facilitator.

